# Association of Dietary Pattern with Cardiovascular Risk Factors among Postmenopausal Women in Taiwan: A Cross-Sectional Study from 2001 to 2015

**DOI:** 10.3390/nu14142911

**Published:** 2022-07-15

**Authors:** Sabrina Aliné, Chien-Yeh Hsu, Hsiu-An Lee, Rathi Paramastri, Jane C.-J. Chao

**Affiliations:** 1 School of Nutrition and Health Sciences, College of Nutrition, Taipei Medical University, 250 Wu-Hsing Street, Taipei 11031, Taiwan; sabrinaline14@gmail.com (S.A.); rara.paramastri@gmail.com (R.P.); 2 Department of Information Management, National Taipei University of Nursing and Health Sciences, 365 Ming-Te Road, Peitou District, Taipei 11219, Taiwan; cyhsu@ntunhs.edu.tw; 3 Master Program in Global Health and Development, College of Public Health, Taipei Medical University, 250 Wu-Hsing Street, Taipei 11031, Taiwan; 4 National Health Research Institutes, 35 Keyan Road, Zhunan Town, Miaoli County 35053, Taiwan; billy72325@gmail.com; 5 Nutrition Research Center, Taipei Medical University Hospital, 252 Wu-Hsing Street, Taipei 11031, Taiwan

**Keywords:** postmenopausal women, dietary pattern, cardiovascular risk factors, reduced rank regression

## Abstract

Unhealthy diet and inappropriate lifestyle contribute to an imbalance in cardiometabolic profiles among postmenopausal women. This research aimed to analyze the association between dietary pattern and changes in cardiovascular risk factors among postmenopausal Taiwanese women using binary logistic regression. This cross-sectional study involved 5689 postmenopausal Taiwanese women aged 45 years and above, and the data were obtained from Mei Jau Health Management Institution database between 2001 and 2015. The cardiovascular risk dietary pattern characterized by high intakes of processed food, rice/flour products, organ meat, and sauce was derived by reduced rank regression. Participants in the highest quartile of the cardiovascular risk dietary pattern were more likely to have high levels of systolic blood pressure (OR = 1.29, 95% CI 1.08–1.53), diastolic blood pressure (OR = 1.28, 95% CI 1.01–1.62), atherogenic index of plasma (OR = 1.26, 95% CI 1.06–1.49), triglycerides (OR = 1.38, 95% CI 1.17–1.62), and fasting blood glucose (Q3: OR = 1.45, 95% CI 1.07–1.97). However, this dietary pattern was not correlated with total cholesterol, low-density lipoprotein cholesterol, high-density lipoprotein cholesterol, and C-reactive protein. Therefore, adherence to the cardiovascular risk dietary pattern increases the risk of having higher levels of blood pressure, triglycerides, fasting blood glucose in postmenopausal Taiwanese women.

## 1. Introduction

Menopause is defined as the cessation of menstruation owing to a decrease in ovarian follicles and the further reduction of estradiol levels. It occurs mostly at a median age of 51 years [1]. The diagnosis of menopause is based on no menstrual period for 12 consecutive months in women [2,3]. Around 467 million postmenopausal women were registered in the world in the 1990s, and by 2030 the number of postmenopausal women is expected to be 1.2 billion with 47 million new postmenopausal women each year [4]. Several studies revealed that postmenopause was associated with increased inflammatory markers such as C-reactive protein (CRP), interleukin-1α (IL-1α), and tumor necrosis factor-α (TNF-α) and an imbalance in cardiometabolic profiles such as low levels of high-density lipoprotein cholesterol (HDL-C) and elevated levels of total cholesterol (TC), low-density lipoprotein cholesterol (LDL-C), triglycerides (TG), visceral fat, and blood glucose [5,6,7,8]. These imbalanced cardiometabolic profiles were favorable for the progression of atherosclerosis and an increased risk of cardiovascular disease (CVD) [9]. Cardiovascular disease was known as the leading cause of mortality worldwide between 1990 and 2019. This scourge claimed around 18.6 million individuals’ lives in 2019 [10]. According to the report by the Ministry of Health and Welfare, Taiwan, heart disease was the second leading cause of death following malignant neoplasms in 2020 [11]. Shen et al. found that in Taiwanese women, early age at menopause between 45 and 49 years was linked to higher CVD death rate and all-cause mortality [12]. In 2018, the percentage of the elderly aged 65 years and above in Taiwan surpassed 14% and has become an aged society [13]. The prevalence of cardiovascular disease, diabetes, and cancer was high among the Taiwanese elderly during the past decade [14]. Aging and atherosclerosis can cause vascular wall damage and estrogen receptor loss, and a decrease in circulating estrogen also reduces estrogen receptors in both vascular endothelium and vascular smooth muscle cells [3]. Additionally, women with vasomotor symptoms have significantly higher blood pressure, elevated circulating total cholesterol levels, and greater body mass index (BMI) than women without vasomotor symptoms [3].

Evidence showed that postmenopausal Chinese women increased the risk of dyslipidemia after multiple adjustment as compared to premenopausal women probably due to the loss of endogenous estrogen after menopause [15]. Some studies also supported that menopause was associated with adverse changes of cardiometabolic profiles and increased risk and mortality of CVD [5,15,16]. Research conducted by Lin et al. demonstrated that compared to premenopausal women in North Taiwan, postmenopausal women had considerably greater odds of having central obesity, metabolic syndrome, high blood pressure, and high blood triglycerides [17]. In addition, diet has been associated with cardiovascular risk factors and other health-related outcomes. A healthy balanced diet plays a significant role in the prevention and mortality reduction of chronic diseases [18]. However, postmenopausal women consuming an unhealthy diet such as high intake of sodium, added sugar, trans fats, and red meat but low intake of fruit, whole grains, fibers, fish, nuts, and legumes were correlated with abnormal fasting blood glucose, high BMI, hypertension, and high blood cholesterol which are considered as risk factors of CVD among postmenopausal women [19]. Brazilian postmenopausal women who consumed a low-quality diet with an excessive intake of sodium and low intakes of vegetables and fruit had central obesity, higher blood pressure, and increased levels of blood lipids and fasting blood glucose [20].

The dietary pattern is considered as a new approach applied in nutritional epidemiology to assess the relationship between dietary factors and disease risk [21]. However, little is known about the outcomes resulting from the association between dietary patterns and CVD risk factors among postmenopausal Taiwanese women. Hence, the aim of this study was to analyze the association between dietary patterns and changes in cardiovascular risk factors such as blood pressure, blood lipids, blood glucose, and CRP among postmenopausal Taiwanese women.

## 2. Materials and Methods

### 2.1. Study Population and Data Source

**Figure 1:**
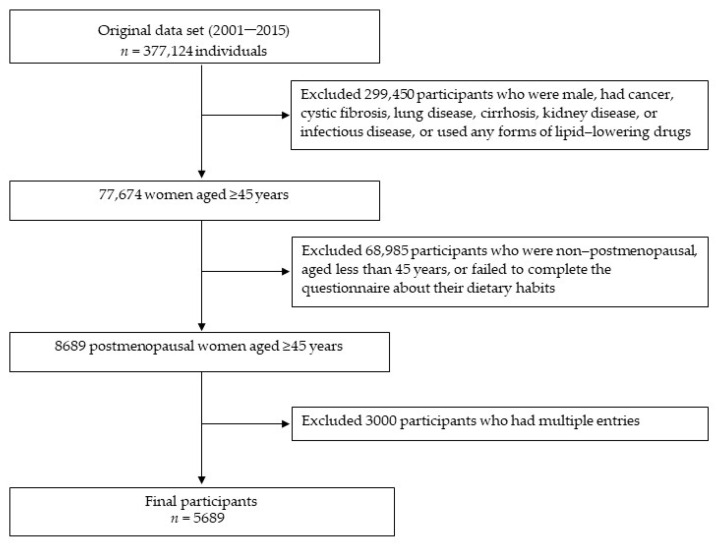
Flowchart of study participants.

This cross-sectional study was conducted using the database from 2001 to 2015, and the data were collected by the Mei Jau (MJ) Health Screening Centers which are located in Taipei, Taoyuan, Taichung, and Kaohsiung cities in Taiwan. All the subjects signed the consent form and agreed their data only for research use without their identity before their health check-up at the MJ Health Screening Center. While visiting the MJ Health Screening Center, all the subjects filled the questionnaires to collect information about their socio-demographic status, lifestyle, and dietary habits by the self-reported questionnaires. Blood samples were analyzed for biochemical parameters. The study was approved by the Joint Institutional Review Board of Taipei Medical University (TMU-JIRB N202007075). There were 377,124 subjects who visited the MJ Health Screening Center between 2005 and 2015. We included women aged ≥45 years who self-reported menopausal status after missing their menstrual period for at least 12 consecutive months using a questionnaire. We excluded 299,450 participants who were male, had disease conditions such as cancer, cystic fibrosis, lung disease, cirrhosis, kidney disease, or infectious disease, or used any forms of lipid-lowering drugs. In addition, we excluded 68,985 women who were non-postmenopausal, aged less than 45 years, or failed to complete the questionnaire about their dietary habits. After excluding 3000 participants who had multiple entries between 2005 and 2015, a total 5689 postmenopausal women were retained in this study (Figure 1).

### 2.2. Dietary Assessment and Other Covariates

A semi-quantitative food frequency questionnaire (FFQ) was developed, standardized, and validated by the MJ Health Management Institution, and used to assess dietary habits of the subjects. The FFQ questionnaire contained the closed-ended questions about 22 non-overlapping food groups with a total of 85 individual food items consumed by the participants in the past month [22]. The intake frequency was assessed in accordance with daily and weekly consumption. Each question was given the definition about one serving size of the food item, and presented 5 frequency response options as described previously [22]. Dietary data were collected for further frequency response options as described previously [22]. Dietary data were collected for further analysis to derive the dietary pattern using a reduced rank regression (RRR) model. The RRR model as a multivariable linear function was performed to derive the dietary pattern related to the disease of interest by a priori and a posteriori approaches based on the response variables for identifying a linear combination of the predictor variables [23].

Demographic data such as age, education (≤high school or >high school), and marital status (never married, married, or divorced/widowed) were collected. We also evaluated lifestyle data including smoking status (no or yes), drinking alcohol (no or yes), physical activity frequency (<150 min/week or ≥150 min/week), and sleep duration (<6 h, 6–8 h, or >8 h). Medical history regarding hypertension, diabetes mellitus, and CVD was recorded. All covariates were assessed using a self-reported questionnaire.

### 2.3. Anthropometric, Clinical, and Biochemical Data

Anthropometric parameters such as height, weight, waist circumference (WC), and waist-to-hip ratio (WHR) were assessed using an anthropometer with electronic scale at the MJ Health Screening Center. The values of BMI were calculated using weight (kg) divided by height (m^2^) [24]. To identify central obesity among the participants, WC (≥80 cm) [25] and WHR (≥0.85) [26] were measured and calculated. Blood pressure was measured twice at 10 min intervals using a standardized sphygmomanometer. Biochemical data such as total cholesterol (TC), low-density lipoprotein cholesterol (LDL-C), high-density lipoprotein cholesterol (HDL-C), triglycerides (TG), fasting blood glucose (FBG), and C-reactive protein (CRP) were assessed after overnight fasting for 12–14 h by the central laboratory at the MJ Health Management Institution. Blood TC, HDL-C, TG, and FBG were evaluated using the commercial kits (Randox Laboratories Ltd., Antrim, UK). The levels of LDL-C were determined by Friedewald formula (LDL-C (mg/dL) = TC-HDL-C-TG/5) [27]. Atherogenic index of plasma (AIP) as an indicator for CVD risk was calculated by the following formula: AIP = log(TG/HDL-C) [28]. Inflammatory marker CRP was diagnosed by the reagent from Fortress Diagnostics (Antrim, UK). Cardiovascular disease risk factors were defined as: systolic blood pressure (SBP) ≥140 mmHg and/or diastolic blood pressure (DBP) ≥ 90 mmHg [20,28], AIP ≥ 0.24 with high risk of CVD [29], TC ≥ 5.17 mmol/L (200 mg/dL) [20], LDL-C ≥ 2.59 mmol/L (100 mg/dL) [20], HDL-C ≤ 1.29 mmol/L (50 mg/dL) [20], TG ≥ 1.69 mmol/L (150 mg/dL) [20], FBG ≥ 7.0 mmol/L (126 mg/dL) [28], and CRP ≥ 28.6 nmol/L (3 mg/L) [30].

### 2.4. Statistical Analysis

**Figure 2:**
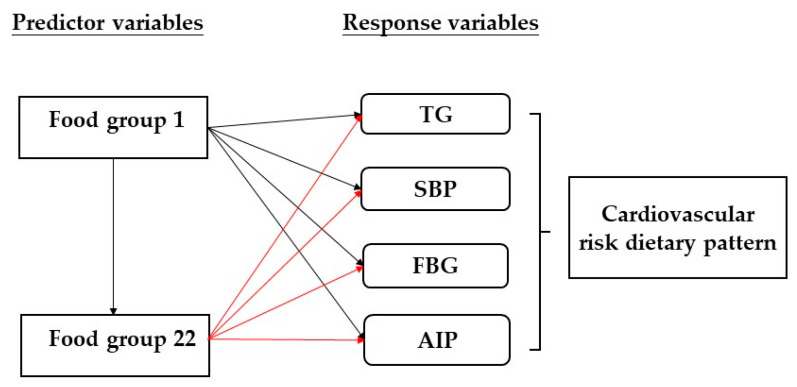
Cardiovascular risk dietary pattern derived from reduced rank regression model. TG: triglycerides, SBP: systolic blood pressure, FBG: fasting blood glucose, AIP: atherogenic index of plasma.

Statistical analysis was performed using SAS version 9.4 (SAS Institute Inc., Chicago, IL, USA) and IBM SPSS 20 (IBM Corp., Armonk, NY, USA). Kolmogorov–Smirnov test was used to determine the distribution of the data. To compare the differences between two groups, Mann–Whitney U test and chi-square test were used for categorical data. To compare data among multiple groups, one-way analysis of variance (ANOVA) and Kruskal–Wallis test were performed. We used binary logistic regression expressed as odds ratios (ORs) and 95% confidence intervals (CIs) to determine the association between the dietary pattern and cardiovascular risk factors. The dietary pattern was derived by RRR using PROC PLS function in SAS 9.4, and 22 food groups were considered as the predictors. After performing Pearson’s correlation coefficient, triglycerides, systolic blood pressure, fasting blood glucose, and AIP were retained as the response variables (Figure 2). In compliance with previous investigation, to obtain the dietary pattern linked to CVD risk, the value of factor loading was set at ≥0.20 [31]. The dietary factor score for each food group was calculated by summing food frequency intake weighed by their factor loadings. Finally, we only retained the first dietary factor for further analysis because it explained the maximum variation of the response variables. The derived dietary pattern was then divided into quartiles according to the dietary factor score. The reference group for the cardiovascular risk dietary pattern was quartile 1 (Q1) which was the lowest quartile of the dietary factor score, and quartile 4 (Q4) represented the highest quartile of the dietary factor score. In binary logistic regression analysis, model 1 was unadjusted, model 2 was adjusted for age, BMI, WC, and WHR, and model 3 was adjusted for model 2 variables plus education, family income, smoking, drinking alcohol, physical activity frequency, and sleep duration. The *p*-value < 0.05 was considered statistically significant.

## 3. Results

### 3.1. Characteristics of Study Participants

Table 1 presents the demographic and lifestyle characteristics of the participants. The majority of postmenopausal women in this study had education below high school (81.5%), non-professional occupation (63.9%), low annual income (<NTD800,000: 69.1%), married status (70.3%), no smoking (97.9%), no drinking alcohol (95.5%), less physical activity frequency (<150 min/week: 55.5%), and sleep duration for 6–8 h (58.6%). The anthropometric, clinical, and biochemical data are shown in Table 2. The majority of postmenopausal women had normal BMI (44.2%), waist circumference (56.1%), and waist-to-hip ratio (68.9%). However, 31.3% postmenopausal women were overweight, 22.7% subjects were obese, 43.9% subjects had central obesity, and 31.1% subjects had abnormal waist-to-hip ratio. The prevalence of hypertension, diabetes, and CVD was 11.3%, 17.5%, and 10.7%, respectively. The mean value of AIP (0.3 ± 0.3) was higher than 0.24 defined as a CVD risk factor. The mean values of TC (5.9 ± 0.8 mmol/L) and LDL-C (3.7 ± 0.8 mmol/L) were abnormal among postmenopausal women. Among 5689 participants, only 7.3% subjects had normal FBG level (<7.0 mmol/L, data not shown).

### 3.2. Cardiovascular Risk Dietary Pattern

**Figure 3:**
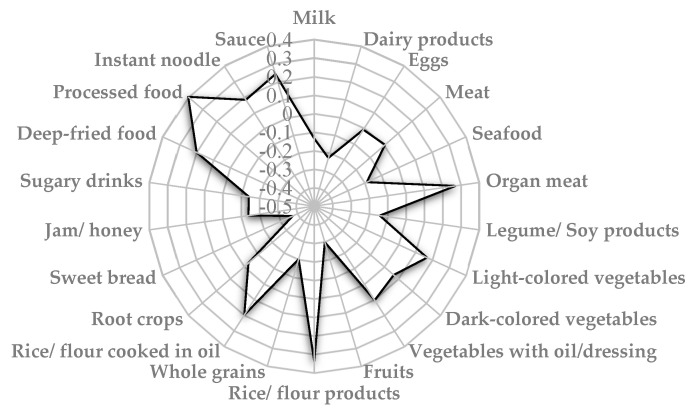
Spider-web diagram of factor loadings for cardiovascular risk dietary pattern.

A dietary pattern identified as a “cardiovascular risk dietary pattern” was derived using the RRR model. Four food groups including processed food, rice/flour products, organ meat, and sauce showed a positive correlation (factor loading ≥ 0.20) with the cardiovascular risk dietary pattern, meanwhile food groups such as dairy products, fruits, whole grains, and sweet bread had a negative correlation with this dietary pattern (factor loading ≤ −0.20) (Figure 3). The cardiovascular risk dietary pattern explained 6.6% cumulative percentage of variation and 1.7% of the total variation for the four response variables. The explained variation was 1.5% for TG, 1.6% for AIP, and 1.8% for both SBP and FBG.

### 3.3. Association between the Dietary Pattern and Cardiovascular Risk Factors

The unadjusted and adjusted models for the association of the cardiovascular risk dietary pattern with SBP, DBP, and AIP among postmenopausal Taiwanese women are presented in Table 3. Model 1 was unadjusted, model 2 was adjusted for age, BMI, WC, and WHR, and model 3 was adjusted for model 2 variables plus education, family income, smoking, drinking alcohol, physical activity frequency, and sleep duration. The results showed that participants in the higher quartiles (Q3 and Q4) of the cardiovascular risk dietary pattern were more likely to increase the odds of having high SBP (OR = 1.40–1.84), high DBP (OR = 1.28–1.69), and high AIP (OR = 1.43–1.69) compared to those in the reference group (Q1) before adjustment. After adjusting variables in models 2 and 3, participants in the highest quartile (Q4) of the cardiovascular risk dietary pattern were still more likely to increase the odds of having high SBP (model 2: OR = 1.42, 95% CI 1.20–1.68, model 3: OR = 1.29, 95% CI 1.08–1.53), high DBP (model 2: OR = 1.43, 95% CI 1.13–1.79, model 3: OR = 1.28, 95% CI 1.01–1.62), and high AIP (model 2: OR = 1.29, 95% CI 1.09–1.52, model 3: OR = 1.26, 95% CI 1.06–1.49).

The association of the cardiovascular risk dietary pattern with TC, LDL-C, and HDL-C among postmenopausal Taiwanese women in the unadjusted and adjusted models is shown in Table 4. The cardiovascular risk dietary pattern was not correlated with the odds of high TC in all models. Participants in the Q2 quartile of the cardiovascular risk dietary pattern were more likely to decrease the odds of having high LDL-C in all models (model 1: OR = 0.63, 95% CI 0.47–0.83, model 2: OR = 0.68, 95% CI 0.51–0.91, model 3: OR = 0.71, 95% CI 0.53–0.94) compared to those in the Q1 quartile. Participants in the higher quartiles (Q3 and Q4) of the cardiovascular risk dietary pattern were more likely to decrease the odds of having low HDL-C in the unadjusted model; however, no association was found after adjustment in models 2 and 3.

Table 5 demonstrates the association of the cardiovascular risk dietary pattern with TG, FBG, and CRP in the unadjusted and adjusted models among postmenopausal Taiwanese women. Participants in the higher quartiles (Q2–Q4) of the cardiovascular risk dietary pattern were more likely to increase the odds of having high TG in all models (model 1: OR = 1.39–1.79, model 2: OR = 1.21–1.43, model 3: OR = 1.18–1.38) compared to those in the lowest quartile (Q1). Participants in the higher quartile (Q3) of the cardiovascular risk dietary pattern were more likely to increase the odds of having high FBG in all models (model 1: OR = 1.75, 95% CI 1.30–2.35, model 2: OR = 1.54, 95% CI 1.14–2.07, model 3: OR = 1.45, 95% CI 1.07–1.97). Participants in the higher quartiles (Q3 and Q4) of the cardiovascular risk dietary pattern were more likely to increase the odds of having high CRP (Q3: OR = 1.38, 95% CI 1.13–1.67, Q4: OR = 1.51, 95% CI 1.25–1.83) only in the unadjusted model.

## 4. Discussion

### 4.1. Association between the Dietary Pattern and Cardiovascular Risk Factors

In this cross-sectional study of 5689 postmenopausal Taiwanese women, we derived the cardiovascular risk dietary pattern and found a positive association with several CVD risk factors such as SBP, DBP, AIP, TG, and FBG. Among the participants in the highest quartile of the cardiovascular risk dietary pattern, 60.4% of postmenopausal women were overweight or obese, and 64.6% were physically inactive (<150 min/week) (data not shown). The cardiovascular risk dietary pattern was recognized by high consumption of processed food, rice/flour products, organ meat, and sauce, but low intakes of dairy products, fruit, whole grains, and sweet bread. The cardiovascular risk dietary pattern reflected similar characteristics as the western dietary pattern recognized by high intakes of processed food, meat, organ meat, rice/flour products, but low consumption of fruit, dark-colored vegetables, bread, and legume/soy products among Taiwanese middle-aged and elderly with chronic kidney disease [23]. Processed food and organ meat are often rich in calories, cholesterol, and/or saturated fat, and all of which could contribute to excessive energy consumption [23].

Low fiber and excessive salt and/or sugar in processed food as well as unbalanced saturated and unsaturated fats in animal food could be correlated with abnormal blood pressure, blood lipids, and blood glucose among Taiwanese middle-aged adults and elderly [23]. Highly refined carbohydrate in rice/flour products, a dietary component for high intake in the cardiovascular risk dietary pattern, could be associated with increases in cardiovascular risk and the development of atherosclerosis among middle-aged adults [32].

Our results revealed that the cardiovascular risk dietary pattern was positively associated with blood pressure. We found that the prevalence of hypertension was only 11.3% among 5689 postmenopausal Taiwanese women. Unlike our results, the previous studies conducted among postmenopausal women reported that the prevalence of hypertension was 31.6% and 56.0% in Brazilian and Chinese postmenopausal women, respectively [20,33]. Weight gain and increased sensitivity to salt in the diet might occur due to hormonal changes after menopause and age-associated metabolic changes, which could lead to a raise in blood pressure [34]. Weight status and physical activity could also contribute to abnormal blood pressure. Postmenopausal women aged <65 years with overweight (33.3%) or obesity (42.9%) also had higher prevalence of high blood pressure (130 mmHg/85 mmHg) compared to those who had normal weight (18.8%), and those who did not do aerobic exercise tended to have higher prevalence of high blood pressure compared to those who did aerobic exercise actively (44.0% vs. 14.3%, *p* = 0.06) [20].

Our findings showed that the cardiovascular risk dietary pattern was correlated with an increase in AIP among postmenopausal women. Numerous studies demonstrated that AIP was an important cardiovascular risk factor and a better predictor for CVD [33,35,36]. The previous studies have reported that AIP was a better predictor of the fractional esterification rate of HDL-C which is a powerful predictor of CVD [35], and a more sensitive diagnostic marker for studies of CVD [35], and a more sensitive diagnostic marker for CVD among postmenopausal women compared to traditional lipid parameters [35,36].

Our results revealed that participants in the highest quartile (Q4) of the cardiovascular risk dietary pattern were more likely to increase the odds of having high CRP before adjustment, even the association between dietary pattern and CRP was not significant after adjustment. A previous study conducted in Southern Brazil among postmenopausal women also observed that participants with high CRP were positively correlated with BMI, WC, body fat, TG, glucose, sedentary lifestyle, and excessive dietary carbohydrate intake (>55% of total energy) [37].

Although the association between aberrant lipid profiles and certain nutrients or food groups has been established, few have demonstrated the association between dietary pattern or quality and blood lipids in postmenopausal women [32]. We found that the cardiovascular risk dietary pattern was positively correlated with increased odds of high TG among postmenopausal women after full adjustment. Brazilian postmenopausal women with a low-quality inadequate diet characterized by an excessive intake of sodium (>2400 mg/day) had increases in the prevalence of high TC and high LDL-C known as cardiovascular risk factors [20]. However, Tardivo and co-workers [32] showed that there was no significant association between diet quality determined by healthy eating index scores and blood lipids in Brazilian postmenopausal women. A study conducted among Korean women showed that postmenopausal women who consumed the western dietary pattern with high intakes of oil and fats, meat, eggs, fast food, and sweets but low intake of grains were correlated with hyper LDL-C [38]. Other studies conducted among Chinese women and Japanese women consuming a western dietary pattern with high intakes of milk, dairy products, and fast food but a low intake of rice or vegetables revealed an imbalance in lipid profile, especially increases in TC and LDL-C [39,40]. The abnormality of various serum lipids was linked to hormonal changes, such as the rise in circulating androgen and the reduction in estrogen, during the menopausal transition period [39].

Adherence to a western type dietary pattern could be associated with the status of being overweight or obese and having high WC, which might contribute to metabolic alteration. The metabolic changes in postmenopausal women could explain the imbalance of CVD-related biochemical variables [41]. Because of estrogen deficiency, postmenopausal women could increase CVD risk factors including central obesity, elevated blood pressure, increased blood lipids, decreased glucose tolerance, and increased vascular inflammation [42]. Compared to premenopausal women, postmenopausal women were more prone to increase blood lipids, which could lead to increase the risk for the development of atherogenesis [16]. In addition, the dietary components could be correlated with abnormal CVD-related biochemical variables in postmenopausal women. High consumption of energy [43], saturated fatty acids [44,45], trans fats [45], cholesterol [46], and eggs [46] was associated with an increased risk of CVD or abnormal CVD-related biochemical variables among postmenopausal women. In contrast, a low-fat dietary pattern [45] or the dietary pattern with high consumption of plant food such as whole grains, vegetables, fruits, legumes, and nuts or seeds, but low intakes of processed food, red meat, sugar, and sodium [47] were correlated with a reduced risk of CVD among postmenopausal women. The cardiovascular risk dietary pattern identified in our study was characterized by high intakes of processed food, rice/flour products, organ meat, and sauce which were accompanied by a high amount of energy, saturated fats, trans fats, cholesterol, added sugar, and sodium. Although the underlying mechanism for the effects of dietary patterns or dietary components on CVD risk factors among postmenopausal women has not been fully understood, changes in lipid metabolism and the increased accumulation of visceral fat related to estrogen deficiency in postmenopausal women could partially contribute to the effect of the dietary pattern on CVD risk factors.

### 4.2. Strengths and Limitations

To our knowledge, the present study is the first one to identify the cardiovascular risk dietary pattern in postmenopausal Taiwanese women using the RRR model as a novel and powerful method. Additionally, the RRR model gave more explanation about the association between the dietary pattern and the disease of interest. Since the RRR-derived dietary pattern was generated by a disease-specific response, the response variables were correlated to the disease of concern [48]. Instead of explaining the variation in significant biomarkers, principal component analysis only provided the explanation of the overall variation in food group intake [48]. Meanwhile, by maximizing the explained variation in the biomarkers for diet-related disorders, the RRR model could be able to predict dietary pattern scores. Researchers can also determine the percentage variance using the RRR approach from the predictor variables and response variables, and both of which contributed to the dietary component [48]. Both the corresponding response scores and the explained variation in the predictor variables could be used to evaluate the extracted factor scores [48]. The large study population collected for 15 years could be representative of postmenopausal Taiwanese women. We also included demographic, anthropometric, clinical, biochemical, and dietary data to explore the association between these variables. However, a number of methodological limitations need to be addressed. First, our study was a cross-sectional study which provided features of eating habits and other characteristics at a specific time point and could raise the possibility of reverse causation bias. Second, the information for FFQ used to identify dietary habits could have self-reported bias. Additionally, the FFQ could be used for an estimate of habitual food intake but not for actual nutrient consumption. Even though the analysis was adjusted for the majority of known confounding variables, the residual confounding bias due to unknown or unmeasured covariates could not be completely ruled out. A longitudinal study is needed to explore the association between dietary patterns and CVD risk factors among postmenopausal Taiwanese women. Further research should be conducted to compare the association in premenopausal versus postmenopausal Taiwanese women.

## 5. Conclusions

The cardiovascular risk dietary pattern with a high intake of processed food, rice/flour products, organ meat, and sauce is associated with increased odds of high blood pressure, AIP, TG, and FBG among postmenopausal women. Our study suggests that choosing a healthier dietary pattern with a lower intake of processed food, rice/flour products, organ meat, and sauce could reduce the risk of CVD in postmenopausal Taiwanese women.

## Figures and Tables

**Table 1:** Demographic and lifestyle characteristics of postmenopausal women aged ≥45 years (*n* = 5689) ^1^.

Variables	Participants (*n* = 5689)
Age (years)	60.6 ± 7.6
Education	
<High school	4636 (81.5)
≥High school	1053 (18.5)
Occupation	
Non-professional	3637 (63.9)
Professional	1269 (22.3)
Unemployed/retired	783 (13.8)
Annual family income (NTD)	
<800,000	3929 (69.1)
810,000–1,600,000	1347 (23.7)
>1,600,000	413 (7.2)
Marital status	
Never married	83 (1.5)
Married	4002 (70.3)
Widows/divorced	1604 (28.2)
Smoking	
No	5567 (97.9)
Yes	122 (2.1)
Drinking alcohol	
No	5433 (95.5)
Yes	256 (4.5)
Physical activity frequency	
<150 min/week	3160 (55.5)
≥150 min/week	2529 (44.5)
Sleep duration	
<6 h	1917 (33.7)
6–8 h	3333 (58.6)
>8 h	439 (7.7)

^1^ Continuous data are presented as mean ± SD and categorical data are expressed as numbers (percentage).

**Table 2:** Demographic, clinical, and biochemical data of postmenopausal women aged ≥45 years (*n* = 5689) ^1^.

Variables	Participants (*n* = 5689)
Body mass index (kg/m^2^)	
<18.5	100 (1.8)
18.5–23.9	2516 (44.2)
24–26.9	1780 (31.3)
≥27	1293 (22.7)
Waist circumference	
<80 cm	3191 (56.1)
≥80 cm	2498 (43.9)
Waist-to-hip ratio	
<0.85	3922 (68.9)
≥0.85	1767 (31.1)
Prevalence of chronic disease	
Hypertension	642 (11.3)
Diabetes mellitus	994 (17.5)
Cardiovascular disease	609 (10.7)
Systolic blood pressure (mmHg)	133 ± 20
Diastolic blood pressure (mmHg)	75 ± 12
Atherogenic index of plasma	0.3 ± 0.3
Total cholesterol (mmol/L)	5.9 ± 0.8
Low-density lipoprotein cholesterol (mmol/L)	3.7 ± 0.8
High-density lipoprotein cholesterol (mmol/L)	1.5 ± 0.4
Triglycerides (mmol/L)	1.6 ± 0.8
Fasting blood glucose (mmol/L)	6.6 ± 1.9
C-reactive protein (nmol/L)	26.8 ± 47.2

^1^ Continuous data are presented as mean ± SD and categorical data are expressed as numbers (percentage).

**Table 3:** Binary logistic regression for the association between the dietary pattern, systolic blood pressure (SBP), diastolic blood pressure (DBP), and atherogenic index of plasma (AIP) (*n* = 5689).

Dietary Pattern	Cardiovascular Disease Risk Factors ^1^		
	High SBP	High DBP	High AIP
	Odds Ratio (95% Confidence Interval)		
Model 1 ^2^			
Q1 (reference)	1	1	1
Q2	1.29 (1.09–1.52) ***	1.22 (0.96–1.54)	1.41 (1.21–1.64) ***
Q3	1.40 (1.19–1.65) ***	1.28 (1.01–1.63) *	1.43 (1.23–1.67) ***
Q4	1.84 (1.58–2.16) ***	1.69 (1.35–2.12) ***	1.69 (1.45–1.98) ***
*p* for trend	0.000	0.000	0.000
Model 2 ^3^			
Q1 (reference)	1	1	1
Q2	1.15 (0.97–1.36)	1.13 (0.89–1.44)	1.29 (1.09–1.51) **
Q3	1.19 (1.00–1.41) *	1.14 (0.89–1.46)	1.18 (1.01–1.39) *
Q4	1.42 (1.20–1.68) ***	1.43 (1.13–1.79) **	1.29 (1.09–1.52) **
*p* for trend	0.000	0.016	0.005
Model 3 ^4^			
Q1 (reference)	1	1	1
Q2	1.09 (0.92–1.29)	1.07 (0.84–1.37)	1.28 (1.09–1.50) **
Q3	1.10 (0.92–1.31)	1.05 (0.82–1.34)	1.16 (0.99–1.37)
Q4	1.29 (1.08–1.53) **	1.28 (1.01–1.62) *	1.26 (1.06–1.49) **
*p* for trend	0.030	0.144	0.013

^1^ High SBP, high DBP, and high AIP were defined as SBP ≥ 140 mmHg, DBP ≥ 90 mmHg, and AIP ≥ 0.24, respectively. ^2^ Model 1 was unadjusted. ^3^ Model 2 was adjusted for age, body mass index, waist circumference, and waist-to-hip ratio. ^4^ Model 3 was adjusted for model 2 variables plus education, family income, smoking, drinking alcohol, physical activity frequency, and sleep duration. * *p* < 0.05, ** *p* < 0.01, *** *p* < 0.001, significantly different from the reference group.

**Table 4:** Binary logistic regression for the association between the dietary pattern, total cholesterol (TC), low-density lipoprotein cholesterol (LDL-C), and high-density lipoprotein cholesterol (HDL-C) (*n* = 5689).

Dietary Pattern	Cardiovascular Disease Risk Factors ^1^		
	High TC	High LDL-C	Low HDL-C
	Odds Ratio (95% Confidence Interval)		
Model 1 ^2^			
Q1 (reference)	1	1	1
Q2	0.84 (0.67–1.05)	0.63 (0.47–0.83) **	0.88 (0.75–1.04)
Q3	0.97 (0.77–1.22)	0.82 (0.60–1.10)	0.81 (0.69–0.96) *
Q4	0.87 (0.70–1.09)	0.78 (0.58–1.05)	0.73 (0.62–0.86) ***
*p* for trend	0.349	0.013	0.002
Model 2 ^3^			
Q1 (reference)	1	1	1
Q2	0.92 (0.73–1.15)	0.68 (0.51–0.91) **	0.95 (0.81–1.13)
Q3	1.11 (0.88–1.39)	0.92 (0.68–1.24)	0.93 (0.79–1.11)
Q4	1.08 (0.86–1.35)	0.94 (0.69–1.27)	0.90 (0.76–1.06)
*p* for trend	0.334	0.022	0.665
Model 3 ^4^			
Q1 (reference)	1	1	1
Q2	0.92 (0.74–1.16)	0.71 (0.53–0.94) *	0.98 (0.83–1.16)
Q3	1.11 (0.87–1.40)	0.99 (0.73–1.35)	0.98 (0.82–1.16)
Q4	1.08 (0.85–1.37)	1.04 (0.77–1.42)	0.96 (0.81–1.14)
*p* for trend	0.381	0.013	0.971

^1^ High TC, high LDL-C, and low HDL-C were defined as TC ≥ 5.17 mmol/L (200 mg/dL), LDL-C ≥ 2.59 mmol/L (100 mg/dL), and HDL-C ≤ 1.29 mmol/L (50 mg/dL), respectively. ^2^ Model 1 was unadjusted. ^3^ Model 2 was adjusted for age, body mass index, waist circumference, and waist-to-hip ratio. ^4^ Model 3 was adjusted for model 2 variables plus education, family income, smoking, drinking alcohol, physical activity frequency, and sleep duration. * *p* < 0.05, ** *p* < 0.01, *** *p* < 0.001, significantly different from the reference group.

**Table 5:** Binary logistic regression for the association between the dietary pattern, triglycerides (TG), fasting blood glucose (FBG), and C-reactive protein (CRP) (*n* = 5689).

Dietary Pattern	Cardiovascular Disease Risk Factors ^1^		
	High TG	High FBG	High CRP
	Odds Ratio (95% Confidence Interval)		
Model 1 ^2^			
Q1 (reference)	1	1	1
Q2	1.39 (1.19–1.63) ***	1.19 (0.92–1.55)	1.12 (0.92–1.37)
Q3	1.42 (1.21–1.66) ***	1.75 (1.30–2.35) ***	1.38 (1.13–1.67) **
Q4	1.79 (1.54–2.09) ***	1.42 (1.08–1.86) *	1.51 (1.25–1.83) ***
*p* for trend	0.000	0.002	0.000
Model 2 ^3^			
Q1 (reference)	1	1	1
Q2	1.29 (1.10–1.51) **	1.10 (0.84–1.43)	0.99 (0.81–1.22)
Q3	1.21 (1.03–1.43) *	1.54 (1.14–2.07) **	1.14 (0.93–1.39)
Q4	1.43 (1.22–1.68) ***	1.16 (0.87–1.53)	1.14 (0.93–1.39)
*p* for trend	0.000	0.040	0.322
Model 3 ^4^			
Q1 (reference)	1	1	1
Q2	1.27 (1.09–1.50) **	1.07 (0.82–1.39)	0.99 (0.80–1.21)
Q3	1.18 (1.00–1.40) *	1.45 (1.07–1.97) *	1.11 (0.90–1.36)
Q4	1.38 (1.17–1.62) ***	1.05 (0.79–1.41)	1.09 (0.89–1.34)
*p* for trend	0.001	0.971	0.569

^1^ High TG, high FBG, and high CRP were defined as TG ≥ 1.69 mmol/L (150 mg/dL), FBG ≥ 7.0 mmol/L (126 mg/dL), and CRP ≥ 28.6 nmol/L (3 mg/L), respectively. ^2^ Model 1 was unadjusted. ^3^ Model 3 was adjusted for age, body mass index, waist circumference, and waist-to-hip ratio. ^4^ Model 4 was adjusted for model 2 variables plus education, family income, smoking, drinking alcohol, physical activity frequency, and sleep duration. * *p* < 0.05, ** *p* < 0.01, *** *p* < 0.001, significantly different from the reference group.

## Data Availability

The data that support the findings of this study are available from the Mei Jau Health Management Institution, but restricted for research use only. The data are not publicly available.
